# *De Novo* Assembly and Characterization of *Oryza officinalis* Leaf Transcriptome by Using RNA-Seq

**DOI:** 10.1155/2015/982065

**Published:** 2015-02-02

**Authors:** Ying Bao, Si Xu, Xiang Jing, Lu Meng, Zongyan Qin

**Affiliations:** School of Life Science, Qufu Normal University, Qufu, Shandong 273165, China

## Abstract

Although endeavors have been made to identify useful wild rice genes that can be used to improve cultivated rice, the virtual reservoir of genetic variation hidden within the wild relatives of cultivated rice is largely untapped. Here, using next-generation sequencing technology, we investigated the leaf transcriptome of a wild rice *O. officinalis* with CC genome. Approximately 23 million reads were produced in the species leaf transcriptome analysis and *de novo* assembly methods constructed 68,132 unigenes. Functional annotations for the unigenes were conducted using sequence similarity comparisons against the following databases: the nonredundant nucleotide database, the nonredundant protein database, the SWISS-PROT database, the Clusters of Orthologous Groups of proteins database, the Kyoto Encyclopedia of Genes and Genomes database, the Gene Ontology Consortium database, and the InterPro domains database. In addition, a total of 476 unigenes related to disease resistance were identified in *O. officinalis*, and these unigenes can serve as important genetic resources for cultivated rice breeding and quality improvement. The present study broadens our understanding of the genetic background of non-AA genomic wild rice species and it also provides a bridge to extend studies to other* Oryza* species with CC genomes.

## 1. Introduction

The transcriptome is defined as “the complete complement of mRNA molecules generated by a cell or population of cells” [1, 2]. Accessibility to transcriptomic information allows us to answer long-standing questions regarding the genetic basis of environmental adaptation and intraspecies divergence as well as the evolutionary differences associated with gene expression in plant-specific growth and developmental stages. Although genome-wide scanning and high-throughput sequencing studies have focused on genetic model plants, this situation has dramatically changed with the rapid development of next-generation sequencing technology and the simultaneous maturation of bioinformatic methods [3].* De novo* assembly and characterization of nonmodel plants by RNA-sequencing have become more feasible. Many nonmodel plants such as* Flaveria* [4],* Leymus chinensis* [5], and* Panicum maximum* [6] have been successfully sequenced at the transcriptome level, and some common underlying evolutionary mechanisms have been elucidated through comparative analyses of related species [4, 7, 8].

*Oryza* is a very important agricultural genus that includes rice (*O. sativa* L.), which is a major source of crop that supports approximately half of the world's population [9]. The genus is composed of 23 species, including two cultivated and 21 wild rice species with 10 genome types (AA, BB, CC, BBCC, CCDD, EE, FF, GG, HHJJ, and HHKK) [10, 11]. As the direct gene pool of cultivated rice (AA genome), wild* Oryza* species possess abundant genetic diversity. Traditional hybridization has long been occurred between cultivated rice and AA genome wild rice species [12], and the potential value of non-AA genome species in rice breeding has also been recognized. For example, alleles associated with resistances to bacterial blight, brown planthoppers and white-backed planthoppers from* O. latifolia* Desv. (CCDD genome),* O. officinalis* Wall ex Watt (CC genome), and* O. australiensis* Domin. (EE genome) have been successfully introgressed into cultivated rice* O. sativa* populations [13–15]. However, the virtual reservoir of genetic variation hidden within the wild relatives of cultivated rice is largely untapped.* O. officinalis* is a perennial wild rice that is distributed in South and Southeast Asia, South China, Papua New Guinea, and Australia. The CC genome possessed by* O. officinalis* is shared by three diploids (*O. eichingeri* Peter,* O. officinalis*, and* O. rhizomatis* Vaughan) and six allotetraploids (three for BBCC genomes:* O. malampuzhaensis* Krish. and Chand.,* O. minuta* J.S. Presl. ex. C.B. Presl., and* O. punctata* Kotschy ex Steud.; three for CCDD genomes:* O. alta* Swallen,* O. grandiglumis* (Doell.) Prod., and* O. latifolia* Desv.) [10, 11]. In addition, the CC genome is phylogenetically close to the AA and BB genomes [9–11]. Therefore, further investigation of the genetic basis of* O. officinalis* will not only provide more opportunities to discover valuable genes that may improve the quality of cultivated rice but will also serve as a bridge to extend further study to other allopolyploids or diploids that contain CC genomes.

## 2. Materials and Methods

### 2.1. Plant Materials

Seeds of three biological replicas of* O. officinalis* (Acc. number 104973) from the International Rice Research Institute (IRRI, Manila, Philippines) were dehulled and heated at 50°C for five days to break dormancy and were subsequently immersed in warm water at 30°C for three days to germinate. The germinated seeds were planted in three small pots at 24°C for two weeks, and the seedlings were transplanted into three large pots (30 × 30 cm) in the Qufu Normal University's greenhouse (length of lightening: 12 h; day/night temperature: 28°C/22°C; moisture: 40%) under normal soil. Young flag leaves from each biological replica were harvested 60 days after germination and were mixed together in equal quantities for RNA extraction.

### 2.2. RNA Extraction, Library Construction, Clustering, and Sequencing

Total RNA was extracted from leaf tissues using the Trizol method (Invitrogen). RNA concentration and quality were assessed by analyzing 1 *μ*L of the RNA sample on an Agilent Technology 2100 Bioanalyzer. The RNA library was constructed using a TruSeq RNA Sample Preparation Kit (RS-122-2001, Illumina) according to the manufacturer's protocols. The library was qualified using the Agilent 2100 Bioanalyzer and quantification was conducted using Qubit and qPCR. Cluster formation and sequencing were performed on the HiSeq2000 platform following the manufacturer cBot and sequencing protocols. The library was run on a single lane for 100 cycles (CapitalBio Corporation), and transcriptome reads are available in GenBank with the accession number SRR1582383.

### 2.3. *De Novo* Assembly and Functional Annotation

After trimming adaptor sequences and removing low quality reads (reads with ambiguous bases “N”) and reads with Q < 30 bases, the remaining reads were assembled with Trinity software [16] to construct unique transcripts. Nonredundant unique transcripts were defined as unigenes. The trimmed Solexa transcriptome reads were mapped onto the unigenes using Bowtie2-2.2.3 software (Bowtie parameter: -v 3 –all –best –strata) [17] to detect the genes expression profiles. Functional annotations for the unigenes were conducted by sequence similarity comparisons against the nonredundant nucleotide database and the nonredundant protein database of NCBI (http://www.ncbi.nlm.nih.gov/) with BLASTx (*E* values cutoff ≤ 1*e* ^−5^) as well as the SWISS-PROT database (European Bioinformatics Institute, ftp://ftp.ebi.ac.uk/pub/databases/swissprot/), the Clusters of Orthologous Groups of proteins database (COG) [18, 19], and the Kyoto Encyclopedia of Genes and Genomes database (KEGG) [20] with BLASTx (*E* values cutoff ≤ 1*e* ^−10^). Moreover, functional assignments of the unigenes and InterPro domains [21] were further annotated using Gene Ontology (GO) [22] and InterProScan [23], and functional classifications of the unigenes were identified using WEGO software [24]. In addition, the unigenes were compared to the rice genome (Os-Nipponbare-Reference-IRGSP-1.0) by using BLASTn (*E* values cutoff ≤ 1*e* ^−5^).

## 3. Results 

### 3.1. *De Novo* Assembly of the* O. officinalis* Transcriptome

**Figure 1:**
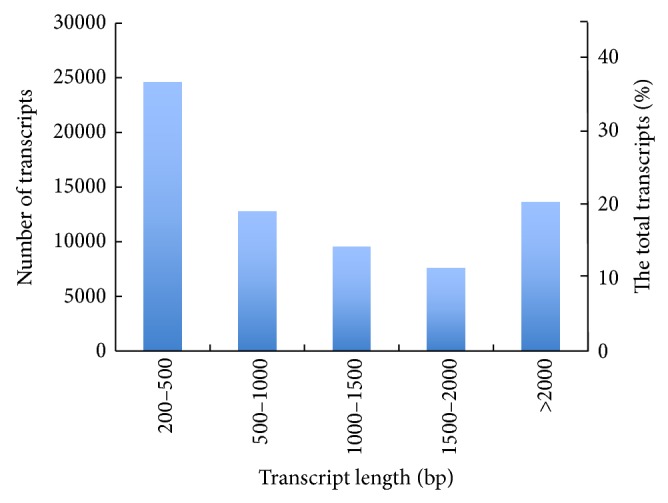
Assemblies of length distribution.

In this study, approximately 23 million high-quality reads with nucleotide sequences totaling 2,131,363,516 bp were obtained, and each read was 100 bp in length. All of the high-quality reads were* de novo* assembled using Trinity software [16], because of the absence of an* O. officinalis* reference genome. The nonredundant assembly resulted in 68,132 unigenes with a total length of 83,266,858 bp and an average length of 1222 bp. The single assembly length ranged from 201 bp to 13,067 bp. The majority of the assemblies (36%) were 200–500 bp, and 20% of the assemblies were longer than 2,000 bp. The remaining assemblies fell into 500–1,000 bp, 1,000–1,500 bp, and 1,000–2,000 bp ranges and represented 19%, 14%, and 11% of the assemblies, respectively (Figure 1). Ninety percent of the 23 million reads were successfully mapped onto the assemblies.

### 3.2. Functional Annotations Using Transcript BLAST Analyses

**Figure 2:**
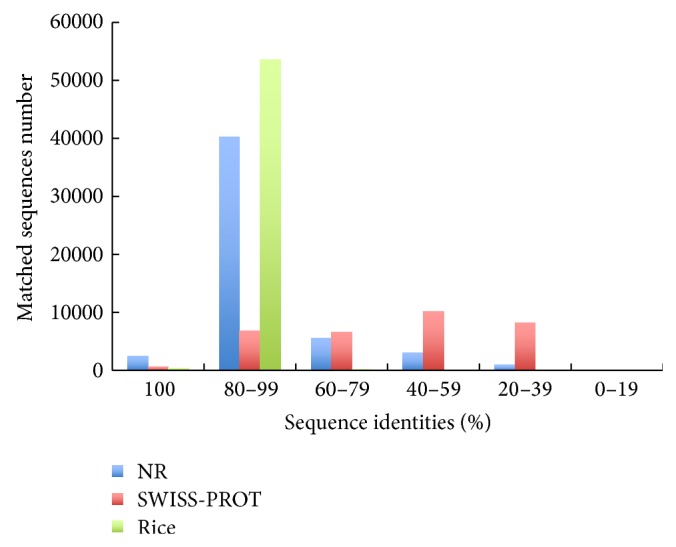
Sequences identities between query and subject sequences by comparing against the nonredundant protein database (NR), the SWISS-PROT database, and Rice genome (Os-Nipponbare-Reference-IRGSP-1.0).

Functional annotations of the unigenes were performed using BLAST comparisons with different databases (Table 1). Of the 68,132 unigenes, significant hits at the nucleotide level were obtained for 65,303 (96%) unigenes using the annotated sequences deposited in the nonredundant nucleotide database (*E* values cutoff ≤ 1*e* ^−5^) (Table 1). The sequences identities were all greater than 77%. Of the matched sequences, 92% were* Oryza* homologs, and 94% were orthologous to the sequences from Poaceae. When comparing protein-coding sequences only, 77% of the unigenes had significant BLAST results for the nonredundant protein database (*E* values cutoff ≤ 1*e* ^−5^); and 48% met a slightly strict standard (*E* values cutoff ≤ 1*e* ^−10^) in the SWISS-PROT protein database (Table 1). Moreover, 42,708 (83%) unigenes had high sequence identities (≥80%) with homologous sequences in the nonredundant protein database, while 7,490 (23%) of unigenes shared high sequences identities (≥80%) with homologous sequences in the SWISS-PROT database (Figure 2). In addition, 54,190 (80%)* O. officinalis* unigenes (Table 1) were perfectly matched to the rice genome (Os-Nipponbare-Reference-IRGSP-1.0) with high sequence identities (>80%) (Figure 2) and the* E*-value = 0 for 60% of the matched unigenes.

### 3.3. Functional Annotation Based on Gene Ontology (GO) and InterPro

**Figure 3:**
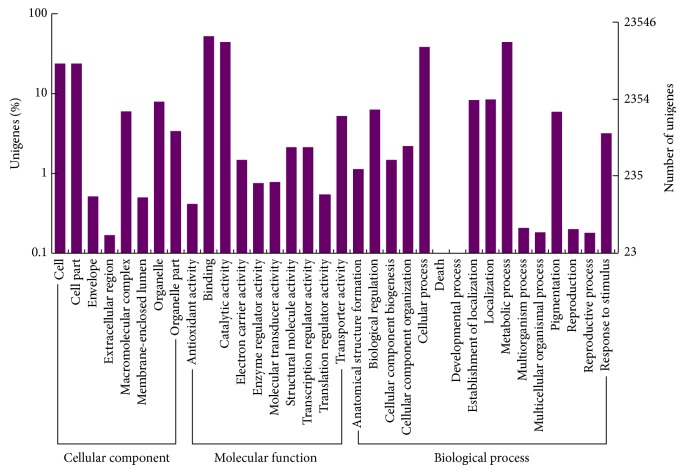
Functional annotation based on Gene Ontology (GO).

Gene ontology (GO) provides a systematic language to describe the attributes of genes and gene products, which includes three key biological domains that are shared by all organisms: molecular function, biological process, and cellular component [25]. In this study, of the 68,132 unigenes, 23,568 (Table 1) were assigned to at least one of the three biological domains (18,364 for molecular function, 30,646 for biological process, and 33,608 for cellular component) and 34 GO subcategories (Figure 3). In addition, a relatively higher proportion of unigenes were grouped into the following GO annotations: “binding (14,485 unigenes),” “catalytic activity (12,396 unigenes),” “metabolic process (12,287 unigenes),” “cellular process (10746 unigenes),” “cell (6,595 unigenes),” and “cell part (6,595 unigenes).”

Protein functions were also predicted by InterPro [21]. Forty-one percent of the unigenes (27,935) were matched to known protein domains (Table 1), and all of the InterPro entries could be mapped to the 34 GO subcategories described above. In addition, based on the above BLAST results and rice functional annotations, we identified a total of 476 unigenes (GenBank accession GBRJ00000000) that were related to disease resistance in the* O. officinalis* leaf transcriptome (see Table S1 in Supplementary Material available online at http://dx.doi.org/10.1155/2015/982065). Of these unigenes, 442 had complete open reading frames, and the codon region lengths of the unigenes varied from 102 bp to 3,774 bp. GC percent of codon regions of the unigenes was at the range of 28%–76%. Different expression levels among the unigenes were also found. There was approximately 1500-fold expression divergence between the highest and the lowest expressed unigenes. Functional annotations showed that the resistance unigenes were all associated with the nucleotide-bing site (NBS) and leucine-rich repeat (LRR) proteins.

### 3.4. COG Classification and KEGG Pathway Mapping

**Figure 4:**
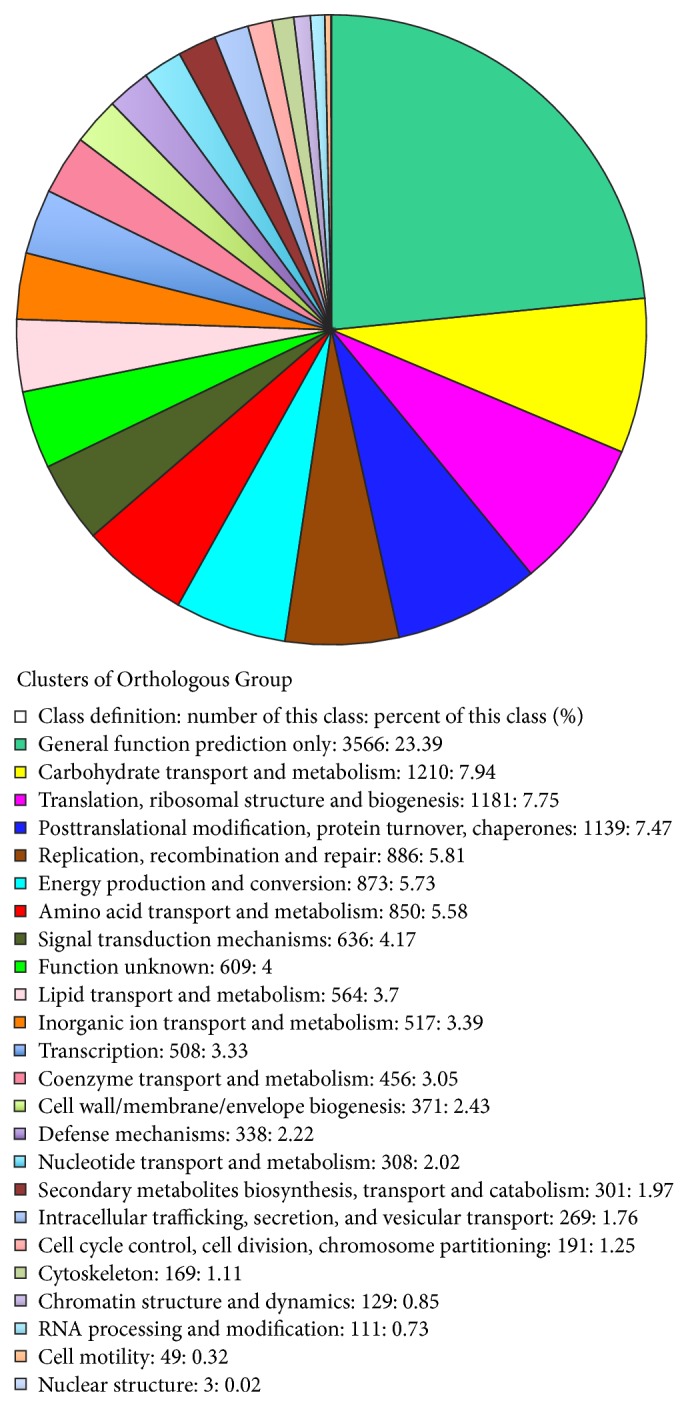
Clusters of Orthologous Group classification.

The COG (Cluster of Orthologous Group) database was designed to classify proteins on the basis of the orthology concept. The database includes 66 unicellular prokaryotic genomes and seven multicellular eukaryotic genomes [18, 19]. Using BLAST searches against these genomes (*E* values cutoff ≤ 1*e* ^−10^), 17,643 (26%) unigenes of the* O. officinalis* leaf transcriptome that showed significant homologous scores (Table 1) were classified into 25 clusters. Among the clusters, the COG “general function predication only” cluster matched the highest proportion (approximately 23%) of unigenes. Additionally, 7.94%, 7.75%, and 7.47% of the unigenes were classified into the “carbohydrate transport and metabolism,” “translation, ribosomal structure and biogenesis,” and “posttranslational modification, protein turnover, chaperones” clusters, respectively. In contrast, only 0.32% and 0.02% of the unigenes were assigned to the “cell motility” and “nuclear structure” clusters, respectively (Figure 4).

**Figure 5:**
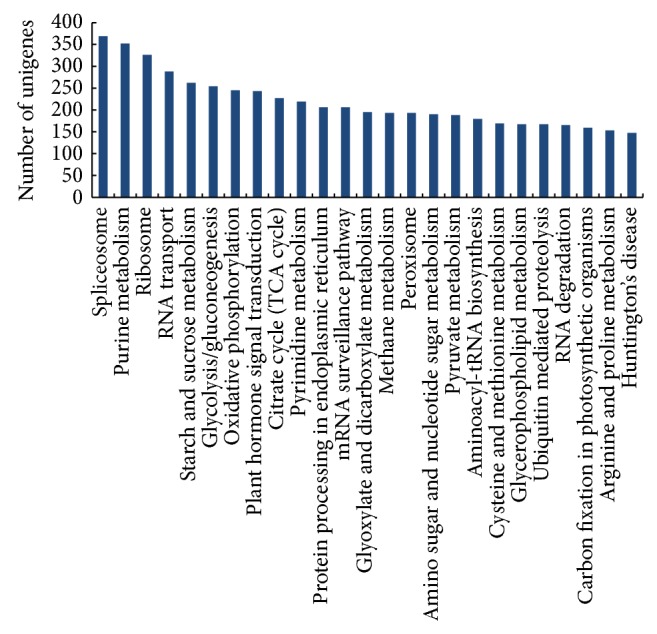
The top 25 mapped pathways annotated by the KEGG database.

Approximately 70% (47,564) of the unigenes were matched to homologs in the KEGG database (Table 1), and 22% (10,476) of those could be mapped to at least one biological pathway. These pathways were mainly associated with five categories, “metabolism” (9,420 unigenes), “genetic information processing” (6,884 unigenes), “environmental information processing” (1,227 unigenes), “cellular processes” (1,300 unigenes), and “human diseases” (1,166 unigenes). The specific pathways, including “spliceosome,” “purine metabolism,” “ribosome,” “RNA transport,” “starch and sucrose metabolism,” and the other 20 top mapped pathways were shown in Figure 5.

## 4. Discussion

The wild relatives of cultivated rice contain abundant genetic diversity that can be used to improve cultivated rice quality and yield. However, comprehensive genetic backgrounds of different* Oryza* species are largely unknown. Fortunately, this status has begun to change, and a series of studies based on genome-wide sequencing have focused on the important agricultural genus. In addition to the greatly improved reference genome of Asian cultivated rice [26, 27], the whole genomes of African cultivated rice (*O. glaberrima* Steud.) [28] and a distant relative of cultivated rice (*O. brachyantha* A. Chev. and Roehr., FF genome) [29] have been published. The complete reference assemblies for some AA genomic wild rice species (*O. barthii* A. Chev.,* O. glumaepatula* Steud., and* O. nivara* S. D. Sharma and Shastry) and a BB genomic wild rice species (*O. punctata* Kotschy ex Steud.) are continuously released and upgraded by the* Oryza* Map Alignment Project (OMAP) and the* Oryza* Genome Evolution (OGE) Project (http://www.genome.arizona.edu/modules/publisher/item.php?itemid=7) before publication. These projects also include the chromosome 3 short-arm assemblies of an additional eight* Oryza* species, including one polyploid, and the outgroup species* Leersia perrieri* (A. Camus) Launert [9]. These works greatly extend our knowledge of the genomes of different* Oryza *species.

**Figure 6:**
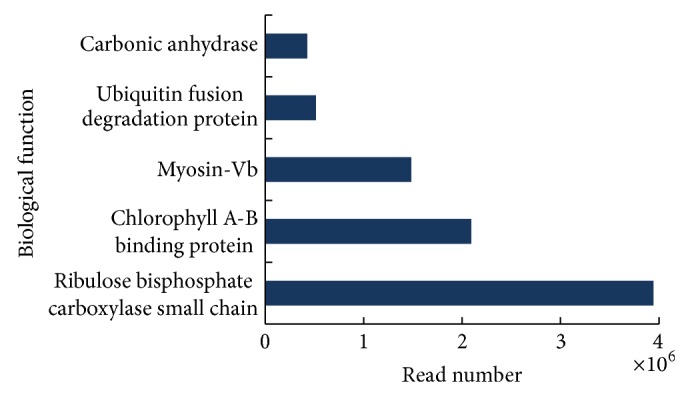
Biological functions of the top five highly expressed unigenes in* O. officinalis* leaf transcriptome.

Here, using next generation sequencing methods, we sequenced the leaf transcriptome of* O. officinalis*, a CC genomic wild rice, and* de novo* assembled 68,132 unigenes based on approximately 23 million transcriptome reads. Although our study only involved one vegetable organ (leaves), this primary gene identification has broadened our understanding of the genetic background of the non-AA genomic wild rice species. For example, of the annotated unigenes, 49% were assigned to cellular components, 45% to biological processes, and 27% to molecular functions (Figure 3). Moreover, four GO subcategory annotations (“binding,” “catalytic activity,” “metabolic process,” and “cellular process”) were assigned to more than 10,000 unigenes, respectively. In addition, organ-specific gene expression patterns were revealed in the* O. officinalis* leaf transcriptome. The top five highly expressed unigenes were related to “ribulose bisphosphate carboxylase small chain,” “chlorophyll A-B binding protein,” “myosin-Vb,” “ubiquitin fusion degradation protein,” and “carbonic anhydrase” (Figure 6). Of these five functions, three (“ribulose bisphosphate carboxylase small chain,” “chlorophyll A-B binding protein,” and “carbonic anhydrase”) are involved in photosynthetic processes. The Cluster of Orthologous Group BLAST analyses and the KEGG pathway mapping further indicated that a majority of leaf transcripts were associated with “carbohydrate transport and metabolism” and “translation, ribosomal structure and biogenesis” (Figure 4) and were included in the pathways of “ribosome,” “starch and sucrose metabolism,” “glycolysis/gluconeogenesis,” and “oxidative phosphorylation.”

Meanwhile, in this study, we also identified and characterized 476 unigenes associated with disease resistance (Table S1), and these unigenes were grouped with an ancient family of encoding proteins with nucleotide-bing sites (NBS) and leucine-rich repeat (LRR) domains. NBS-LRR genes that control resistance to a wide variety of pathogens and pests are one of the largest classes of plant disease resistance genes. In cultivated rice, 581 potential NBS-encoding sequences have been identified from the Nipponbare rice genome, and 100 were predicted to be probable pseudogenes [30]. Given that NBS-LRR genes play very important roles in disease defense, the identified expressed sequences in* O. officnalis* are valuable genetic resources for cultivated rice breeding and quality improvement.

## 5. Conclusion

The present transcriptome analysis provides useful data on expressed genes of* O. officinalis*, with 68,132 unigenes identified. These data are invaluable resources for broadening our understanding of the genetic background of non-AA genomic wild rice species and are potential resources for increasing cultivated rice quality and yield. Furthermore, from an evolutionary point of view, the transcriptome of* O. officinalis* with a CC genome provides a bridge to further study the other two diploids CC genomes (*O. eichingeri* and* O. rhizomatis*) and gene pools, and it is also provides the ability to distinguish the subgenome constitution of six allotetraploids (BBCC genome:* O. malampuzhaensis*,* O. minuta*, and* O. punctata*; CCDD genome:* O. alta*,* O. grandiglumis*, and* O. latifolia*) that contain the CC genome.

## Supplementary Material

Data represent the assembled unigenes of O. officinalis assigned to the functions related to disease resistances by comparison against the rice genome (Os-Nipponbare-Reference-IRGSP-1.0) at an E ≤ 1e-5.

## Figures and Tables

**Table 1:** Functional annotations using transcript BLAST analyses.

Database	Hit unigenes number	Percent (hit/total)
NT	65303	95.8%
NR	52210	76.6%
SWISS-PROT	32410	47.6%
COG	17643	25.9%
KEGG	47564	69.8%
GO	23568	34.6%
InterPro	27935	41.0%
Rice	54190	79.5%

NT: the nonredundant nucleotide database; NR: the nonredundant protein database; SWISS-PROT: SWISS-PROT database; COG: the Clusters of Orthologous Groups of proteins database; KEGG: the Kyoto Encyclopedia of Genes and Genomes database; GO: the Gene Ontology Consortium database; InterPro: InterPro domains database; Rice: Os-Nipponbare-Reference-IRGSP-1.0.
