# Supplementary material for: De Novo Assembly and Characterization of Oryza officinalis Leaf Transcriptome by Using RNA-Seq
Source: Biomed Res Int. 2015 Feb 2;2015:982065. doi: 10.1155/2015/982065 (PMC4332758; doi:10.1155/2015/982065)
Supplement: Supplementary file 1 — Data represent the assembled unigenes of O. officinalis assigned to the functions related to disease resistances by comparison against the rice genome (Os-Nipponbare-Reference-IRGSP-1.0) at an E ≤ 1e-5. [file 982065.f1.pdf]

TABLE S1: The unigenes related to disease resistance in *O. officinalis* leaf transcriptome

**Transcripts**	**Length (bp)**	**Expression**	**ORF**				**Rice genome (Os-Nipponbare-Reference-IRGSP-1.0)**				
			**Start**	**End**	**Length (bp)**	**GC (%)**	**Best hits**	**Identity**	**Score**	**E-value**	**Annotation**
Unigene_1	224	7	122	3	120	42.5	LOC_Os12g17340.1	97	396	1.00E-110	CC-NBS-LRR resistance protein MLA13
Unigene_2	351	15	36	350	315	44.44	LOC_Os12g17340.1	95	232	4.00E-60	CC-NBS-LRR resistance protein MLA13
Unigene_3	4233	1092	4027	563	3465	43.35	LOC_Os12g32670.1	84	2339	0	CC-NBS-LRR resistance protein
Unigene_4	4217	1108	4011	547	3465	43.35	LOC_Os12g32670.1	84	2339	0	CC-NBS-LRR resistance protein
Unigene_5	244	9	198	1	198	39.39	LOC_Os08g16580.1	89	270	1.00E-71	Cf2/Cf5 disease resistance protein
Unigene_6	201	6	43	189	147	39.46	LOC_Os05g41310.1	98	375	1.00E-103	Disease resistance protein RGA2
Unigene_7	224	9	109	222	114	56.14	LOC_Os05g41310.1	96	198	3.00E-50	Disease resistance protein RGA2
Unigene_8	3189	975	178	3189	3012	41.77	LOC_Os02g18000.1	81	868	0	Disease resistance protein RGA2
Unigene_9	234	5	131	21	111	44.14	LOC_Os05g41310.1	97	416	1.00E-116	Disease resistance protein RGA2
Unigene_10	532	13	178	531	354	40.68	LOC_Os05g41310.1	95	848	0	Disease resistance protein RGA2
Unigene_11	955	66	580	200	381	51.44	LOC_Os08g12740.1	98	190	4.00E-47	Disease resistance protein RGA3
Unigene_12	242	6	24	242	219	40.18	LOC_Os12g29280.1	97	440	1.00E-123	Disease resistance protein RGA3
Unigene_13	256	4	59	256	198	40.91	LOC_Os12g29280.1	98	476	1.00E-133	Disease resistance protein RGA3
Unigene_14	1611	398	1479	1	1479	40.43	LOC_Os06g47800.1	90	1909	0	Disease resistance protein RGA3
Unigene_15	257	3	45	200	156	39.1	LOC_Os12g29290.1	95	242	3.00E-63	Disease resistance protein RGA3
Unigene_16	2581	272	69	1889	1821	49.92	LOC_Os05g34230.1	90	2446	0	Disease resistance protein RGA3
Unigene_17	1226	136	1153	2	1152	47.22	LOC_Os11g10770.1	85	208	2.00E-52	Disease resistance protein RGA3
Unigene_18	2883	305	340	2562	2223	40.71	LOC_Os11g10770.1	80	103	2.00E-20	Disease resistance protein RGA3
Unigene_19	517	21	39	272	234	58.12	LOC_Os12g29280.1	97	172	5.00E-42	Disease resistance protein RGA3
Unigene_20	252	10	60	251	192	40.62	LOC_Os01g71114.1	98	472	1.00E-132	Disease resistance protein RGA4

Unigene_21	4478	634	4447	674	3774	43.16	LOC_Os05g31550.1	90	2095	0	Disease resistance protein RGA4
Unigene_22	237	6	223	2	222	54.5	LOC_Os01g71114.1	97	414	1.00E-115	Disease resistance protein RGA4
Unigene_23	255	5	98	199	102	36.27	LOC_Os01g36640.1	96	424	1.00E-118	Disease resistance protein RPM1
Unigene_24	382	12	15	380	366	37.43	LOC_Os09g09490.1	96	662	0	Disease resistance protein RPM1
Unigene_25	485	18	67	483	417	39.33	LOC_Os09g09490.1	96	835	0	Disease resistance protein RPM1
Unigene_26	3216	340	27	2834	2808	51.25	LOC_Os08g09430.1	83	1136	0	Disease resistance protein RPM1
Unigene_27	3405	368	27	2924	2898	51	LOC_Os08g09430.1	83	1136	0	Disease resistance protein RPM1
Unigene_28	435	16	414	238	177	44.63	LOC_Os09g09490.1	97	234	1.00E-60	Disease resistance protein RPM1
Unigene_29	207	5					LOC_Os09g09490.1	95	337	5.00E-92	Disease resistance protein RPM1
Unigene_30	638	27	510	1	510	40.98	LOC_Os09g09490.1	97	1146	0	Disease resistance protein RPM1
Unigene_31	483	31	315	482	168	44.64	LOC_Os11g12330.1	84	89.7	5.00E-17	Disease resistance protein RPM1
Unigene_32	644	42	559	128	432	47.92	LOC_Os11g12340.2	82	77.8	3.00E-13	Disease resistance protein RPM1
Unigene_33	1811	92	1740	1	1740	45.92	LOC_Os11g11950.1	79	117	9.00E-25	Disease resistance protein RPM1
Unigene_34	213	5					LOC_Os11g12320.1	98	391	1.00E-108	Disease resistance protein RPM1
Unigene_35	2308	172	1768	2	1767	42.9	LOC_Os12g31620.1	78	280	1.00E-73	Disease resistance protein RPM1
Unigene_36	226	9	49	225	177	36.16	LOC_Os12g31620.1	83	54	1.00E-06	Disease resistance protein RPM1
Unigene_37	269	6	169	62	108	38.89	LOC_Os09g30220.1	95	448	1.00E-125	Disease resistance protein RPM1
Unigene_38	260	7	57	260	204	40.2	LOC_Os07g08890.1	92	351	5.00E-96	Disease resistance protein RPM1
Unigene_39	433	10	5	430	426	41.08	LOC_Os07g08890.1	96	733	0	Disease resistance protein RPM1
Unigene_40	475	14	376	56	321	46.73	LOC_Os08g07774.2	89	58	2.00E-07	Disease resistance protein RPM1
Unigene_41	801	43	178	2	177	44.63	LOC_Os08g32890.1	97	375	1.00E-102	Disease resistance protein RPM1
Unigene_42	279	6	270	1	270	42.59	LOC_Os04g21890.1	96	482	1.00E-135	Disease resistance protein RPM1
Unigene_43	282	8	146	280	135	37.04	LOC_Os04g21890.1	95	456	1.00E-127	Disease resistance protein RPM1
Unigene_44	583	14	295	2	294	46.94	LOC_Os11g11770.1	91	747	0	Disease resistance protein RPM1
Unigene_45	245	7					LOC_Os11g11770.1	96	414	1.00E-115	Disease resistance protein RPM1

Unigene_46	849	30	554	3	552	44.57	LOC_Os11g11770.1	94	1314	0	Disease resistance protein RPM1
Unigene_47	762	39	31	594	564	45.92	LOC_Os11g11770.1	94	954	0	Disease resistance protein RPM1
Unigene_48	775	34	180	413	234	48.72	LOC_Os10g21400.1	83	105	1.00E-21	Disease resistance protein RPM1
Unigene_49	334	15	65	334	270	54.81	LOC_Os07g08890.1	92	438	1.00E-122	Disease resistance protein RPM1
Unigene_50	216	8	185	3	183	50.82	LOC_Os07g08890.1	93	303	8.00E-82	Disease resistance protein RPM1
Unigene_51	1159	80	143	1159	1017	40.81	LOC_Os10g07534.1	82	58	4.00E-07	Disease resistance protein RPM1
Unigene_52	1009	68	143	1009	867	41.41	LOC_Os10g07534.1	82	58	4.00E-07	Disease resistance protein RPM1
Unigene_53	367	18	101	367	267	46.44	LOC_Os01g36640.1	82	77.8	1.00E-13	Disease resistance protein RPM1
Unigene_54	398	14	72	263	192	50	LOC_Os11g12330.1	93	587	1.00E-167	Disease resistance protein RPM1
Unigene_55	730	32	68	730	663	45.25	LOC_Os11g12340.2	96	1233	0	Disease resistance protein RPM1
Unigene_56	583	20	389	583	195	47.18	LOC_Os11g12340.2	95	617	1.00E-175	Disease resistance protein RPM1
Unigene_57	2426	177	2358	397	1962	41.59	LOC_Os02g09790.1	87	2272	0	Disease resistance protein RPM1
Unigene_58	388	19	270	1	270	40.37	LOC_Os02g09790.1	85	307	9.00E-83	Disease resistance protein RPM1
Unigene_59	262	11					LOC_Os02g09790.1	87	65.9	4.00E-10	Disease resistance protein RPM1
Unigene_60	2373	197	2147	3	2145	46.06	LOC_Os10g21400.1	86	343	1.00E-92	Disease resistance protein RPM1
Unigene_61	2316	189	2147	3	2145	46.06	LOC_Os10g21400.1	86	343	1.00E-92	Disease resistance protein RPM1
Unigene_62	2282	512	2202	244	1959	42.16	LOC_Os11g41170.1	96	2789	0	Disease resistance protein RPM1
Unigene_63	3656	685	3117	244	2874	43.7	LOC_Os11g41170.1	96	2789	0	Disease resistance protein RPM1
Unigene_64	3524	672	3117	244	2874	43.7	LOC_Os11g41170.1	96	2789	0	Disease resistance protein RPM1
Unigene_65	817	62	51	608	558	47.31	LOC_Os11g11950.1	95	422	1.00E-117	Disease resistance protein RPM1
Unigene_66	793	70	85	792	708	42.8	LOC_Os11g11950.1	95	1277	0	Disease resistance protein RPM1
Unigene_67	2075	298	160	1182	1023	43.21	LOC_Os11g11960.1	95	2791	0	Disease resistance protein RPM1
Unigene_68	1304	73	347	1303	957	42.22	LOC_Os11g11950.1	97	1076	0	Disease resistance protein RPM1
Unigene_69	3437	806	501	3269	2769	43.91	LOC_Os10g07978.2	94	4605	0	Disease resistance protein RPM1
Unigene_70	3564	810	628	3396	2769	43.91	LOC_Os10g07978.1	93	5134	0	Disease resistance protein RPM1

Unigene_71	3958	832	1022	3790	2769	43.91	LOC_Os10g07978.1	93	5134	0	Disease resistance protein RPM1
Unigene_72	3831	828	895	3663	2769	43.91	LOC_Os10g07978.2	94	4605	0	Disease resistance protein RPM1
Unigene_73	4833	1920	307	3276	2970	43.3	LOC_Os06g17900.1	92	3610	0	Disease resistance protein RPM1
Unigene_74	5370	1982	985	3813	2829	42.38	LOC_Os06g17900.1	92	3610	0	Disease resistance protein RPM1
Unigene_75	4817	1939	985	3813	2829	42.38	LOC_Os06g17900.1	92	3610	0	Disease resistance protein RPM1
Unigene_76	4280	1877	307	3276	2970	43.3	LOC_Os06g17900.1	92	3610	0	Disease resistance protein RPM1
Unigene_77	580	121	194	580	387	74.16	LOC_Os08g28470.1	90	83.8	4.00E-15	Disease resistance protein RPM1
Unigene_78	752	29	81	752	672	40.33	LOC_Os09g30220.1	96	1308	0	Disease resistance protein RPM1
Unigene_79	435	13	301	2	300	34.67	LOC_Os09g30220.1	97	767	0	Disease resistance protein RPM1
Unigene_80	240	5					LOC_Os09g30220.1	95	383	1.00E-105	Disease resistance protein RPM1
Unigene_81	305	9					LOC_Os11g12340.2	90	357	9.00E-98	Disease resistance protein RPM1
Unigene_82	301	8	141	37	105	27.62	LOC_Os11g12330.1	87	71.9	7.00E-12	Disease resistance protein RPM1
Unigene_83	256	7	24	161	138	52.9	LOC_Os11g12330.1	92	232	3.00E-60	Disease resistance protein RPM1
Unigene_84	215	4	122	3	120	43.33	LOC_Os09g30220.1	88	198	3.00E-50	Disease resistance protein RPM1
Unigene_85	361	9	170	361	192	44.27	LOC_Os08g32890.1	98	365	1.00E-100	Disease resistance protein RPM1
Unigene_86	456	16	158	3	156	42.31	LOC_Os07g08890.1	95	731	0	Disease resistance protein RPM1
Unigene_87	230	10	206	3	204	58.82	LOC_Os01g57870.1	98	426	1.00E-119	Disease resistance protein RPS2
Unigene_88	207	6					LOC_Os01g57870.1	97	363	1.00E-100	Disease resistance protein RPS2
Unigene_89	261	6	92	247	156	41.03	LOC_Os01g57870.1	91	278	6.00E-74	Disease resistance protein RPS2
Unigene_90	246	5	7	246	240	58.33	LOC_Os01g57870.1	98	464	1.00E-130	Disease resistance protein RPS2
Unigene_91	247	13	20	184	165	51.52	LOC_Os01g57870.1	96	418	1.00E-116	Disease resistance protein RPS2
Unigene_92	282	15	19	282	264	45.45	LOC_Os01g57870.1	96	472	1.00E-132	Disease resistance protein RPS2
Unigene_93	568	20	11	568	558	43.55	LOC_Os01g57870.1	95	900	0	Disease resistance protein RPS2
Unigene_94	224	6	172	2	171	38.6	LOC_Os12g30720.1	98	412	1.00E-114	Disease resistance protein RPS2
Unigene_95	315	7	194	3	192	46.35	LOC_Os01g57870.1	99	601	1.00E-171	Disease resistance protein RPS2

Unigene_96	850	25	83	793	711	40.65	LOC_Os01g06836.1	89	440	1.00E-122	Disease resistance protein slve2 precursor
Unigene_97	429	11	289	143	147	36.05	LOC_Os01g06836.1	83	184	1.00E-45	Disease resistance protein slve2 precursor
Unigene_98	312	9	216	1	216	43.98	LOC_Os07g33720.1	92	248	6.00E-65	Disease resistance protein
Unigene_99	224	7	213	1	213	47.89	LOC_Os07g17220.2	97	396	1.00E-110	Disease resistance protein
Unigene_100	365	8	42	365	324	43.21	LOC_Os07g17220.2	96	636	0	Disease resistance protein
Unigene_101	1018	39	178	855	678	44.69	LOC_Os07g17220.2	92	1308	0	Disease resistance protein
Unigene_102	447	42	244	77	168	55.95	LOC_Os04g03180.1	91	157	2.00E-37	Disease resistance protein
Unigene_103	2154	122	97	1146	1050	40.38	LOC_Os01g58520.1	95	3394	0	Disease resistance protein
Unigene_104	343	7					LOC_Os01g06790.1	83	170	1.00E-41	Disease resistance protein
Unigene_105	429	18	326	207	120	56.67	LOC_Os01g06790.1	83	99.6	5.00E-20	Disease resistance protein
Unigene_106	359	7	16	357	342	40.35	LOC_Os12g30760.1	96	624	1.00E-178	Disease resistance protein
Unigene_107	237	5	219	79	141	35.46	LOC_Os12g39620.2	82	91.7	6.00E-18	Disease resistance protein
Unigene_108	204	10	24	203	180	52.22	LOC_Os03g36920.1	97	365	1.00E-100	Disease resistance protein
Unigene_109	1942	139	61	1806	1746	45.53	LOC_Os03g36920.1	97	3366	0	Disease resistance protein
Unigene_110	206	6					LOC_Os03g36920.1	98	167	1.00E-40	Disease resistance protein
Unigene_111	374	13	207	1	207	42.51	LOC_Os03g36920.1	96	646	0	Disease resistance protein
Unigene_112	788	31	786	1	786	47.33	LOC_Os03g36920.1	97	1386	0	Disease resistance protein
Unigene_113	775	451	400	263	138	51.45	LOC_Os04g03180.1	91	121	2.00E-26	Disease resistance protein
Unigene_114	806	480	159	1	159	52.83	LOC_Os04g03180.1	88	115	2.00E-24	Disease resistance protein
Unigene_115	228	8	80	226	147	43.54	LOC_Os12g30760.1	97	398	1.00E-110	Disease resistance protein
Unigene_116	4722	909	4154	417	3738	45.45	LOC_Os03g63240.1	90	2753	0	Disease resistance protein
Unigene_117	4885	1021	4317	559	3759	45.68	LOC_Os03g63240.1	90	2753	0	Disease resistance protein
Unigene_118	4794	1031	4226	468	3759	45.68	LOC_Os03g63240.1	90	2753	0	Disease resistance protein
Unigene_119	703	34	122	469	348	46.55	LOC_Os11g12350.1	94	714	0	Disease resistance protein
Unigene_120	4793	4629	4651	2069	2583	58.15	LOC_Os12g39620.3	94	4440	0	Disease resistance protein

Unigene_121	1008	237	866	12	855	73.33	LOC_Os12g39620.1	93	638	0	Disease resistance protein
Unigene_122	4561	4543	4419	1837	2583	58.15	LOC_Os12g39620.3	94	4440	0	Disease resistance protein
Unigene_123	605	21	447	88	360	54.72	LOC_Os05g50790.1	89	129	8.00E-29	Disease resistance protein
Unigene_124	229	6					LOC_Os01g58520.1	92	252	3.00E-66	Disease resistance protein
Unigene_125	202	5					LOC_Os01g58520.1	92	153	2.00E-36	Disease resistance protein
Unigene_126	1865	159	404	1657	1254	41.31	LOC_Os04g53050.1	84	749	0	Disease resistance RPP13-like protein 1
Unigene_127	286	6	232	2	231	41.13	LOC_Os05g41290.1	92	188	4.00E-47	Disease resistance RPP13-like protein 1
Unigene_128	504	19	382	504	123	43.9	LOC_Os01g57270.1	86	297	1.00E-79	Disease resistance RPP13-like protein 1
Unigene_129	458	13	7	456	450	40.89	LOC_Os05g30220.1	93	640	0	Disease resistance RPP13-like protein 1
Unigene_130	293	6	7	291	285	43.16	LOC_Os05g30220.1	92	283	1.00E-75	Disease resistance RPP13-like protein 1
Unigene_131	565	33	145	564	420	42.38	LOC_Os05g41290.1	96	985	0	Disease resistance RPP13-like protein 1
Unigene_132	749	32	369	1	369	44.72	LOC_Os05g41290.1	94	1072	0	Disease resistance RPP13-like protein 1
Unigene_133	1531	103	94	1380	1287	43.51	LOC_Os05g30220.1	89	1695	0	Disease resistance RPP13-like protein 1
Unigene_134	515	28	10	513	504	39.09	LOC_Os05g30220.1	93	763	0	Disease resistance RPP13-like protein 1
Unigene_135	467	26	289	11	279	49.46	LOC_Os05g30220.1	91	236	3.00E-61	Disease resistance RPP13-like protein 1
Unigene_136	473	11	188	394	207	36.23	LOC_Os10g04090.1	94	58	2.00E-07	Disease resistance RPP13-like protein 1
Unigene_137	1804	90	1417	101	1317	44.8	LOC_Os03g38330.1	95	2997	0	Disease resistance RPP13-like protein 1
Unigene_138	203	7					LOC_Os03g38330.1	97	363	1.00E-100	Disease resistance RPP13-like protein 1
Unigene_139	490	18	61	489	429	39.63	LOC_Os03g38330.1	95	805	0	Disease resistance RPP13-like protein 1
Unigene_140	317	11	35	316	282	44.33	LOC_Os03g38330.1	97	553	1.00E-157	Disease resistance RPP13-like protein 1
Unigene_141	218	6	24	218	195	53.85	LOC_Os05g30220.1	92	295	2.00E-79	Disease resistance RPP13-like protein 1
Unigene_142	212	6	199	71	129	55.81	LOC_Os05g30220.1	92	95.6	4.00E-19	Disease resistance RPP13-like protein 1
Unigene_143	834	35	599	832	234	44.87	LOC_Os01g57270.1	81	379	1.00E-104	Disease resistance RPP13-like protein 1
Unigene_144	213	6	70	213	144	48.61	LOC_Os01g57270.1	89	178	3.00E-44	Disease resistance RPP13-like protein 1
Unigene_145	211	5	28	138	111	45.95	LOC_Os01g57270.1	85	67.9	8.00E-11	Disease resistance RPP13-like protein 1

Unigene_146	393	11	163	2	162	58.02	LOC_Os01g57270.1	85	133	3.00E-30	Disease resistance RPP13-like protein 1
Unigene_147	508	13	179	508	330	46.97	LOC_Os12g32790.1	88	54	3.00E-06	Disease resistance RPP13-like protein 1
Unigene_148	272	5	250	2	249	52.21	LOC_Os10g04090.1	90	307	6.00E-83	Disease resistance RPP13-like protein 1
Unigene_149	210	1	210	1	210	50.95	LOC_Os10g04090.1	90	258	5.00E-68	Disease resistance RPP13-like protein 1
Unigene_150	879	90	510	355	156	34.62	LOC_Os10g04090.1	95	63.9	5.00E-09	Disease resistance RPP13-like protein 1
Unigene_151	2986	327	2889	1216	1674	45.46	LOC_Os04g43340.1	97	4732	0	Disease resistance RPP13-like protein 1
Unigene_152	1912	281	1162	326	837	42.41	LOC_Os04g43340.1	96	1403	0	Disease resistance RPP13-like protein 1
Unigene_153	1456	79	1359	274	1086	47.79	LOC_Os04g43340.1	97	2087	0	Disease resistance RPP13-like protein 1
Unigene_154	1738	248	1209	259	951	43.32	LOC_Os04g43340.1	97	2218	0	Disease resistance RPP13-like protein 1
Unigene_155	1991	286	1462	326	1137	42.74	LOC_Os04g43340.1	96	2355	0	Disease resistance RPP13-like protein 1
Unigene_156	2907	322	2810	1137	1674	45.46	LOC_Os04g43340.1	97	3372	0	Disease resistance RPP13-like protein 1
Unigene_157	3160	360	3063	1390	1674	45.46	LOC_Os04g43340.1	97	3372	0	Disease resistance RPP13-like protein 1
Unigene_158	1659	243	909	259	651	43.16	LOC_Os04g43340.1	97	1267	0	Disease resistance RPP13-like protein 1
Unigene_159	3239	365	3142	1469	1674	45.46	LOC_Os04g43340.1	96	4869	0	Disease resistance RPP13-like protein 1
Unigene_160	422	20	414	97	318	68.24	LOC_Os04g43340.1	92	406	1.00E-112	Disease resistance RPP13-like protein 1
Unigene_161	452	20					LOC_Os10g04090.1	85	58	2.00E-07	Disease resistance RPP13-like protein 1
Unigene_162	339	10					LOC_Os09g20030.1	82	127	2.00E-28	Disease resistance RPP13-like protein 1
Unigene_163	654	33	245	652	408	65.2	LOC_Os03g48370.1	87	105	1.00E-21	Disease resistance RPP13-like protein 1
Unigene_164	994	42	695	3	693	45.89	LOC_Os05g41290.1	95	1245	0	Disease resistance RPP13-like protein 1
Unigene_165	668	16	232	666	435	40.46	LOC_Os10g04090.1	89	773	0	Disease resistance RPP13-like protein 1
Unigene_166	1859	114	125	1585	1461	43.53	LOC_Os09g20030.1	91	2143	0	Disease resistance RPP13-like protein 1
Unigene_167	521	22	21	521	501	42.51	LOC_Os03g20840.1	97	934	0	Disease resistance RPP13-like protein 1
Unigene_168	779	56	680	3	678	67.7	LOC_Os02g47230.1	96	1176	0	Disease resistance/zinc finger/chromosome condensation-like region protein
Unigene_169	535	37	210	422	213	70.42	LOC_Os02g47230.1	95	771	0	Disease resistance/zinc finger/chromosome

											condensation-like region protein
Unigene_170	1383	133	91	1077	987	45.49	LOC_Os03g11340.1	95	2268	0	Leucine-rich repeat resistance protein
Unigene_171	1030	72	22	984	963	49.12	LOC_Os02g16070.1	84	325	1.00E-87	Lr1 disease resistance protein
Unigene_172	429	23	345	1	345	43.77	LOC_Os02g16070.1	86	359	3.00E-98	Lr1 disease resistance protein
Unigene_173	1818	295	93	1619	1527	47.68	LOC_Os12g25170.1	92	2440	0	NB-ARC domain containing disease resistance protein
Unigene_174	217	16					LOC_Os02g10580.1	90	232	2.00E-60	NB-ARC domain containing disease resistance protein
Unigene_175	3135	730	384	1460	1077	67.69	LOC_Os02g10580.1	91	2008	0	NB-ARC domain containing disease resistance protein
Unigene_176	2967	720	384	1460	1077	67.69	LOC_Os02g10580.1	91	2008	0	NB-ARC domain containing disease resistance protein
Unigene_177	2893	750	384	1460	1077	67.69	LOC_Os02g10580.1	91	2008	0	NB-ARC domain containing disease resistance protein
Unigene_178	3159	679	384	1460	1077	67.69	LOC_Os02g10580.1	91	2008	0	NB-ARC domain containing disease resistance protein
Unigene_179	2725	740	384	1460	1077	67.69	LOC_Os02g10580.1	91	2008	0	NB-ARC domain containing disease resistance protein
Unigene_180	2917	699	384	1460	1077	67.69	LOC_Os02g10580.1	91	2008	0	NB-ARC domain containing disease resistance protein
Unigene_181	2991	669	384	1460	1077	67.69	LOC_Os02g10580.1	91	2008	0	NB-ARC domain containing disease resistance protein
Unigene_182	2749	689	384	1460	1077	67.69	LOC_Os02g10580.1	91	2008	0	NB-ARC domain containing disease resistance protein
Unigene_183	513	15	147	512	366	69.95	LOC_Os03g14900.1	97	898	0	NB-ARC/LRR disease resistance protein

Unigene_184	205	10	7	204	198	62.12	LOC_Os03g14900.1	95	335	2.00E-91	NB-ARC/LRR disease resistance protein
Unigene_185	330	10	39	329	291	66.67	LOC_Os03g14900.1	96	551	1.00E-156	NB-ARC/LRR disease resistance protein
Unigene_186	247	9	242	3	240	61.67	LOC_Os03g14900.1	94	387	1.00E-107	NB-ARC/LRR disease resistance protein
Unigene_187	843	47	12	704	693	72.01	LOC_Os03g14900.1	94	1352	0	NB-ARC/LRR disease resistance protein
Unigene_188	3617	478	2996	261	2736	41.52	LOC_Os01g58530.1	88	1857	0	NB-ARC/LRR disease resistance protein
Unigene_189	1263	121	812	132	681	67.4	LOC_Os03g14900.1	94	311	2.00E-83	NB-ARC/LRR disease resistance protein
Unigene_190	1292	123	812	132	681	67.4	LOC_Os03g14900.1	92	636	0	NB-ARC/LRR disease resistance protein
Unigene_191	636	29	395	3	393	43	LOC_Os06g20050.1	94	944	0	NBS type disease resistance protein
Unigene_192	306	7	113	304	192	43.23	LOC_Os06g20050.1	79	61.9	7.00E-09	NBS type disease resistance protein
Unigene_193	245	8					LOC_Os06g20050.1	93	355	3.00E-97	NBS type disease resistance protein
Unigene_194	920	46	142	360	219	41.55	LOC_Os02g18140.1	86	63.9	6.00E-09	NBS type disease resistance protein
Unigene_195	739	35	70	738	669	38.71	LOC_Os06g20050.1	92	1003	0	NBS type disease resistance protein
Unigene_196	445	10	311	3	309	39.81	LOC_Os01g72390.1	96	763	0	NBS type disease resistance protein
Unigene_197	458	31	267	1	267	41.2	LOC_Os06g20050.1	98	837	0	NBS type disease resistance protein
Unigene_198	391	15	111	389	279	62.72	LOC_Os04g53160.1	92	254	1.00E-66	NBS-LRR disease resistance protein
Unigene_199	274	7	5	274	270	38.89	LOC_Os02g02670.1	83	194	7.00E-49	NBS-LRR disease resistance protein
Unigene_200	288	6					LOC_Os02g02640.1	89	333	1.00E-90	NBS-LRR disease resistance protein
Unigene_201	333	11	317	3	315	41.9	LOC_Os02g02670.1	88	228	6.00E-59	NBS-LRR disease resistance protein
Unigene_202	570	23	64	570	507	46.75	LOC_Os04g52970.1	96	858	0	NBS-LRR disease resistance protein
Unigene_203	415	11	76	414	339	38.64	LOC_Os06g49380.3	90	476	1.00E-133	NBS-LRR disease resistance protein
Unigene_204	509	20					LOC_Os12g29710.1	95	165	1.00E-39	NBS-LRR disease resistance protein
Unigene_205	470	20	236	469	234	38.03	LOC_Os09g15840.1	95	331	8.00E-90	NBS-LRR disease resistance protein
Unigene_206	432	17	419	3	417	44.36	LOC_Os01g71106.2	93	630	1.00E-180	NBS-LRR disease resistance protein
Unigene_207	3497	503	105	2942	2838	44.15	LOC_Os08g10260.1	94	4276	0	NBS-LRR disease resistance protein
Unigene_208	238	4	214	2	213	44.13	LOC_Os07g29820.1	96	392	1.00E-108	NBS-LRR disease resistance protein

Unigene_209	1758	98	147	1574	1428	44.89	LOC_Os12g09240.1	83	153	2.00E-35	NBS-LRR disease resistance protein
Unigene_210	730	40	174	626	453	41.06	LOC_Os12g09240.1	79	161	3.00E-38	NBS-LRR disease resistance protein
Unigene_211	491	17	273	1	273	47.99	LOC_Os09g34150.1	96	555	1.00E-157	NBS-LRR disease resistance protein
Unigene_212	474	12	289	2	288	35.42	LOC_Os04g52970.1	91	615	1.00E-175	NBS-LRR disease resistance protein
Unigene_213	245	6					LOC_Os04g52970.1	96	422	1.00E-117	NBS-LRR disease resistance protein
Unigene_214	861	40	565	861	297	48.82	LOC_Os04g53000.1	91	575	1.00E-163	NBS-LRR disease resistance protein
Unigene_215	220	11	138	28	111	48.65	LOC_Os04g53000.1	90	248	4.00E-65	NBS-LRR disease resistance protein
Unigene_216	288	6	17	286	270	45.93	LOC_Os04g52970.1	91	234	8.00E-61	NBS-LRR disease resistance protein
Unigene_217	729	32	50	484	435	54.71	LOC_Os06g49380.4	94	864	0	NBS-LRR disease resistance protein
Unigene_218	290	6	105	1	105	46.67	LOC_Os08g16450.1	92	58	1.00E-07	NBS-LRR disease resistance protein
Unigene_219	276	6	136	276	141	39.72	LOC_Os06g17910.1	85	285	2.00E-76	NBS-LRR disease resistance protein
Unigene_220	216	5	168	37	132	60.61	LOC_Os11g39160.1	92	54	1.00E-06	NBS-LRR disease resistance protein
Unigene_221	281	6	228	106	123	36.59	LOC_Os10g25487.1	91	319	2.00E-86	NBS-LRR disease resistance protein
Unigene_222	1082	56	230	1081	852	42.14	LOC_Os08g19980.1	96	1826	0	NBS-LRR disease resistance protein
Unigene_223	234	7	36	233	198	37.37	LOC_Os08g19980.1	97	408	1.00E-113	NBS-LRR disease resistance protein
Unigene_224	420	21	390	94	297	39.06	LOC_Os08g19980.1	93	609	1.00E-173	NBS-LRR disease resistance protein
Unigene_225	686	65	63	422	360	60.28	LOC_Os08g19980.1	97	581	1.00E-165	NBS-LRR disease resistance protein
Unigene_226	972	58	319	627	309	57.61	LOC_Os01g71106.2	94	557	1.00E-157	NBS-LRR disease resistance protein
Unigene_227	682	27	432	1	432	44.44	LOC_Os01g71106.2	94	1061	0	NBS-LRR disease resistance protein
Unigene_228	541	20	29	541	513	49.12	LOC_Os01g71106.2	92	755	0	NBS-LRR disease resistance protein
Unigene_229	934	43	927	94	834	40.77	LOC_Os01g71106.2	97	1398	0	NBS-LRR disease resistance protein
Unigene_230	285	11	167	3	165	38.18	LOC_Os01g71106.2	97	502	1.00E-141	NBS-LRR disease resistance protein
Unigene_231	412	23	31	324	294	46.94	LOC_Os10g25487.1	93	496	1.00E-139	NBS-LRR disease resistance protein
Unigene_232	656	38	31	324	294	46.94	LOC_Os10g25487.1	93	500	1.00E-140	NBS-LRR disease resistance protein
Unigene_233	256	13	29	256	228	47.81	LOC_Os10g25487.1	92	357	7.00E-98	NBS-LRR disease resistance protein

Unigene_234	592	28	225	590	366	49.45	LOC_Os10g25487.1	93	355	7.00E-97	NBS-LRR disease resistance protein
Unigene_235	739	55	306	1	306	42.48	LOC_Os10g25487.1	96	1235	0	NBS-LRR disease resistance protein
Unigene_236	221	4	94	213	120	40	LOC_Os10g25487.1	91	206	1.00E-52	NBS-LRR disease resistance protein
Unigene_237	1124	76	1087	2	1086	41.71	LOC_Os10g25487.1	95	1810	0	NBS-LRR disease resistance protein
Unigene_238	370	11	182	370	189	58.73	LOC_Os10g25487.1	95	567	1.00E-161	NBS-LRR disease resistance protein
Unigene_239	327	13	9	326	318	46.23	LOC_Os04g53000.1	86	105	6.00E-22	NBS-LRR disease resistance protein
Unigene_240	1081	49	1080	1	1080	43.15	LOC_Os11g29520.1	82	529	1.00E-149	NBS-LRR disease resistance protein
Unigene_241	679	24	637	449	189	42.33	LOC_Os04g53000.1	83	121	2.00E-26	NBS-LRR disease resistance protein
Unigene_242	958	86	25	957	933	41.37	LOC_Os06g49380.3	85	434	1.00E-120	NBS-LRR disease resistance protein
Unigene_243	721	39	710	3	708	37.85	LOC_Os06g49360.1	92	1011	0	NBS-LRR disease resistance protein
Unigene_244	994	46	983	3	981	36.7	LOC_Os06g49380.3	86	898	0	NBS-LRR disease resistance protein
Unigene_245	516	17	20	514	495	64.85	LOC_Os06g49390.1	85	307	1.00E-82	NBS-LRR disease resistance protein
Unigene_246	207	5	70	207	138	56.52	LOC_Os06g49390.1	93	230	9.00E-60	NBS-LRR disease resistance protein
Unigene_247	267	6	30	266	237	62.03	LOC_Os06g49390.1	84	83.8	2.00E-15	NBS-LRR disease resistance protein
Unigene_248	1284	78	1181	3	1179	44.61	LOC_Os04g52970.1	94	54	8.00E-06	NBS-LRR disease resistance protein
Unigene_249	212	10	160	2	159	41.51	LOC_Os06g17880.1	89	238	4.00E-62	NBS-LRR disease resistance protein
Unigene_250	260	5	181	50	132	45.45	LOC_Os06g17880.1	95	73.8	2.00E-12	NBS-LRR disease resistance protein
Unigene_251	2693	142	1666	2691	1026	42.4	LOC_Os06g17880.1	94	4159	0	NBS-LRR disease resistance protein
Unigene_252	535	19	439	2	438	48.86	LOC_Os09g34150.1	94	831	0	NBS-LRR disease resistance protein
Unigene_253	522	24	512	3	510	56.67	LOC_Os11g11810.1	80	141	2.00E-32	NBS-LRR disease resistance protein
Unigene_254	283	13					LOC_Os11g11810.1	81	131	9.00E-30	NBS-LRR disease resistance protein
Unigene_255	1881	145	59	1879	1821	45.85	LOC_Os11g11810.1	81	141	6.00E-32	NBS-LRR disease resistance protein
Unigene_256	474	29	118	2	117	37.61	LOC_Os11g11810.1	85	119	6.00E-26	NBS-LRR disease resistance protein
Unigene_257	2312	102	31	2289	2259	44.05	LOC_Os07g29820.1	93	3366	0	NBS-LRR disease resistance protein
Unigene_258	806	57	3	449	447	43.18	LOC_Os07g29820.1	92	916	0	NBS-LRR disease resistance protein

Unigene_259	804	59	115	447	333	39.64	LOC_Os07g29820.1	93	1160	0	NBS-LRR disease resistance protein
Unigene_260	690	28	532	98	435	44.83	LOC_Os06g49380.3	91	795	0	NBS-LRR disease resistance protein
Unigene_261	2681	305	39	1544	1506	48.27	LOC_Os06g49380.3	93	601	1.00E-170	NBS-LRR disease resistance protein
Unigene_262	2203	247	39	1544	1506	48.27	LOC_Os06g49380.3	93	601	1.00E-170	NBS-LRR disease resistance protein
Unigene_263	2645	309	72	1508	1437	48.5	LOC_Os06g49380.3	88	198	4.00E-49	NBS-LRR disease resistance protein
Unigene_264	2167	251	72	1508	1437	48.5	LOC_Os06g49390.1	87	180	8.00E-44	NBS-LRR disease resistance protein
Unigene_265	324	7	94	324	231	47.62	LOC_Os06g49380.3	89	349	2.00E-95	NBS-LRR disease resistance protein
Unigene_266	277	7	229	62	168	43.45	LOC_Os06g49360.1	91	287	6.00E-77	NBS-LRR disease resistance protein
Unigene_267	1538	80	1478	705	774	37.86	LOC_Os07g04900.1	94	1614	0	NBS-LRR disease resistance protein
Unigene_268	2362	202	2118	1162	957	39.92	LOC_Os07g04900.1	88	1211	0	NBS-LRR disease resistance protein
Unigene_269	1284	103	1282	869	414	50.48	LOC_Os07g04900.1	89	523	1.00E-147	NBS-LRR disease resistance protein
Unigene_270	2598	234	249	578	330	55.15	LOC_Os04g53496.1	87	533	1.00E-150	NBS-LRR disease resistance protein
Unigene_271	2446	230	249	578	330	55.15	LOC_Os04g53496.1	87	533	1.00E-150	NBS-LRR disease resistance protein
Unigene_272	1829	137	249	578	330	55.15	LOC_Os04g53496.1	87	533	1.00E-150	NBS-LRR disease resistance protein
Unigene_273	1700	129	249	578	330	55.15	LOC_Os04g53496.1	87	533	1.00E-150	NBS-LRR disease resistance protein
Unigene_274	2469	226	249	578	330	55.15	LOC_Os04g53496.1	87	533	1.00E-150	NBS-LRR disease resistance protein
Unigene_275	1677	133	249	578	330	55.15	LOC_Os04g53496.1	87	533	1.00E-150	NBS-LRR disease resistance protein
Unigene_276	1193	345	919	653	267	51.69	LOC_Os06g17920.1	89	270	7.00E-71	NBS-LRR disease resistance protein
Unigene_277	2263	511	885	616	270	40.74	LOC_Os06g17920.1	89	270	1.00E-70	NBS-LRR disease resistance protein
Unigene_278	1306	376	1032	616	417	46.52	LOC_Os06g17920.1	89	270	7.00E-71	NBS-LRR disease resistance protein
Unigene_279	1403	411	885	616	270	40.74	LOC_Os06g17920.1	89	270	8.00E-71	NBS-LRR disease resistance protein
Unigene_280	1456	423	938	570	369	42.82	LOC_Os06g17920.1	89	270	8.00E-71	NBS-LRR disease resistance protein
Unigene_281	2316	523	938	570	369	42.82	LOC_Os06g17920.1	89	270	1.00E-70	NBS-LRR disease resistance protein
Unigene_282	1246	357	972	706	267	51.69	LOC_Os06g17920.1	89	270	7.00E-71	NBS-LRR disease resistance protein
Unigene_283	2663	281	120	2342	2223	40.71	LOC_Os11g10760.1	77	69.9	3.00E-10	NBS-LRR disease resistance protein

Unigene_284	2211	111	1057	2211	1155	39.48	LOC_Os04g52970.1	96	1086	0	NBS-LRR disease resistance protein
Unigene_285	513	14	217	513	297	40.74	LOC_Os02g02670.1	87	492	1.00E-138	NBS-LRR disease resistance protein
Unigene_286	265	10	241	2	240	42.92	LOC_Os04g52970.1	91	212	3.00E-54	NBS-LRR disease resistance protein
Unigene_287	2224	115	56	2224	2169	40.71	LOC_Os04g52970.1	96	1086	0	NBS-LRR disease resistance protein
Unigene_288	1266	65	112	1266	1155	39.22	LOC_Os11g29520.1	86	543	1.00E-153	NBS-LRR disease resistance protein
Unigene_289	3672	568	762	3671	2910	45.26	LOC_Os11g11810.1	84	303	1.00E-80	NBS-LRR disease resistance protein
Unigene_290	3536	546	762	2966	2205	45.26	LOC_Os11g11810.1	84	303	1.00E-80	NBS-LRR disease resistance protein
Unigene_291	491	22	455	3	453	49.89	LOC_Os12g09240.1	95	79.8	5.00E-14	NBS-LRR disease resistance protein
Unigene_292	3203	695	2895	214	2682	41.01	LOC_Os10g10360.2	96	5233	0	NBS-LRR disease resistance protein
Unigene_293	3753	819	3445	764	2682	41.01	LOC_Os10g10360.1	96	5323	0	NBS-LRR disease resistance protein
Unigene_294	3058	252	1058	3058	2001	44.68	LOC_Os11g45180.1	90	3655	0	NBS-LRR disease resistance protein
Unigene_295	2441	195	1059	2441	1383	41.58	LOC_Os11g45180.1	91	3108	0	NBS-LRR disease resistance protein
Unigene_296	1552	62	211	1551	1341	39.3	LOC_Os08g15880.1	79	188	2.00E-46	NBS-LRR disease resistance protein
Unigene_297	2440	198	1058	2440	1383	41.58	LOC_Os11g45180.1	91	3100	0	NBS-LRR disease resistance protein
Unigene_298	3575	277	1059	3176	2118	45.33	LOC_Os11g45180.1	90	3683	0	NBS-LRR disease resistance protein
Unigene_299	3911	2060	2666	1092	1575	42.03	LOC_Os06g05359.1	93	3499	0	NBS-LRR disease resistance protein
Unigene_300	4745	2932	3500	1926	1575	42.03	LOC_Os06g05359.1	93	3499	0	NBS-LRR disease resistance protein
Unigene_301	641	64	553	287	267	58.05	LOC_Os11g13940.1	85	63.9	4.00E-09	NBS-LRR disease resistance protein
Unigene_302	3799	364	2429	174	2256	41.89	LOC_Os11g13940.1	92	4101	0	NBS-LRR disease resistance protein
Unigene_303	512	41	479	93	387	57.11	LOC_Os11g39160.1	97	432	1.00E-120	NBS-LRR disease resistance protein
Unigene_304	1370	121	209	1120	912	55.48	LOC_Os11g39160.1	93	965	0	NBS-LRR disease resistance protein
Unigene_305	1281	118	209	796	588	59.86	LOC_Os11g39160.1	97	464	1.00E-129	NBS-LRR disease resistance protein
Unigene_306	1636	524	313	1635	1323	41.8	LOC_Os06g17970.1	92	2187	0	NBS-LRR disease resistance protein
Unigene_307	240	9					LOC_Os06g17970.1	88	95.6	4.00E-19	NBS-LRR disease resistance protein
Unigene_308	1451	490	89	1450	1362	41.85	LOC_Os06g17970.1	90	1707	0	NBS-LRR disease resistance protein

Unigene_309	3220	1259	89	1663	1575	43.49	LOC_Os06g17970.1	90	1897	0	NBS-LRR disease resistance protein
Unigene_310	1724	565	627	1022	396	36.87	LOC_Os06g17950.1	87	432	1.00E-119	NBS-LRR disease resistance protein
Unigene_311	1171	522	408	683	276	45.65	LOC_Os06g17950.1	87	432	1.00E-120	NBS-LRR disease resistance protein
Unigene_312	2667	1216	89	1663	1575	43.49	LOC_Os06g17970.1	90	1897	0	NBS-LRR disease resistance protein
Unigene_313	2316	277	1978	1439	540	51.85	LOC_Os06g17920.1	96	200	1.00E-49	NBS-LRR disease resistance protein
Unigene_314	846	74	419	246	174	60.92	LOC_Os06g17920.1	96	200	4.00E-50	NBS-LRR disease resistance protein
Unigene_315	3280	1210	319	2910	2592	47.3	LOC_Os08g10440.1	87	2617	0	NBS-LRR disease resistance protein
Unigene_316	2669	1134	319	2667	2349	46.53	LOC_Os08g10440.1	87	2395	0	NBS-LRR disease resistance protein
Unigene_317	2465	1069	115	2463	2349	46.53	LOC_Os08g10440.1	87	2395	0	NBS-LRR disease resistance protein
Unigene_318	209	19					LOC_Os08g10430.1	96	129	2.00E-29	NBS-LRR disease resistance protein
Unigene_319	3465	1299	504	3095	2592	47.3	LOC_Os08g10440.1	87	2617	0	NBS-LRR disease resistance protein
Unigene_320	2892	1126	115	2706	2592	47.3	LOC_Os08g10440.1	87	2617	0	NBS-LRR disease resistance protein
Unigene_321	3076	1145	115	2706	2592	47.3	LOC_Os08g10440.1	87	2617	0	NBS-LRR disease resistance protein
Unigene_322	208	16	24	176	153	60.13	LOC_Os08g10440.1	88	170	7.00E-42	NBS-LRR disease resistance protein
Unigene_323	2867	1271	517	2865	2349	46.53	LOC_Os08g10440.1	87	2395	0	NBS-LRR disease resistance protein
Unigene_324	3096	1191	319	2910	2592	47.3	LOC_Os08g10440.1	87	2617	0	NBS-LRR disease resistance protein
Unigene_325	3281	1280	504	3095	2592	47.3	LOC_Os08g10440.1	87	2617	0	NBS-LRR disease resistance protein
Unigene_326	3294	1328	517	3108	2592	47.3	LOC_Os08g10440.1	87	2617	0	NBS-LRR disease resistance protein
Unigene_327	2854	1223	504	2852	2349	46.53	LOC_Os08g10440.1	87	2395	0	NBS-LRR disease resistance protein
Unigene_328	3478	1356	517	3108	2592	47.3	LOC_Os08g10440.1	87	2617	0	NBS-LRR disease resistance protein
Unigene_329	377	12	196	2	195	35.9	LOC_Os11g44960.1	100	54	2.00E-06	NBS-LRR disease resistance protein
Unigene_330	234	7	174	1	174	35.06	LOC_Os06g49380.3	97	416	1.00E-116	NBS-LRR disease resistance protein
Unigene_331	203	6					LOC_Os10g25487.1	88	85.7	3.00E-16	NBS-LRR disease resistance protein
Unigene_332	213	7					LOC_Os08g19980.1	100	422	1.00E-117	NBS-LRR disease resistance protein
Unigene_333	204	4	60	203	144	38.19	LOC_Os08g19980.1	98	369	1.00E-101	NBS-LRR disease resistance protein

Unigene_334	426	10	50	424	375	41.07	LOC_Os08g15880.1	83	91.7	1.00E-17	NBS-LRR disease resistance protein
Unigene_335	236	6	18	236	219	50.23	LOC_Os04g53060.1	87	71.9	6.00E-12	NBS-LRR disease resistance protein
Unigene_336	1320	44	1093	143	951	48.16	LOC_Os11g45050.1	83	125	3.00E-27	NBS-LRR disease resistance protein
Unigene_337	203	4					LOC_Os02g38386.1	88	60	2.00E-08	NBS-LRR disease resistance protein
Unigene_338	768	21	48	446	399	39.85	LOC_Os06g49390.1	83	533	1.00E-150	NBS-LRR disease resistance protein
Unigene_339	332	11	149	331	183	43.72	LOC_Os06g17910.1	94	248	6.00E-65	NBS-LRR disease resistance protein
Unigene_340	212	6					LOC_Os12g29690.1	98	389	1.00E-107	NBS-LRR disease resistance protein
Unigene_341	316	8	76	315	240	68.75	LOC_Os09g34150.1	93	313	1.00E-84	NBS-LRR disease resistance protein
Unigene_342	298	7	278	3	276	50.72	LOC_Os08g16460.1	94	127	1.00E-28	NBS-LRR disease resistance protein
Unigene_343	271	8	56	271	216	40.74	LOC_Os06g49380.3	97	466	1.00E-130	NBS-LRR disease resistance protein
Unigene_344	337	9	32	337	306	39.54	LOC_Os11g15670.1	87	321	5.00E-87	NBS-LRR disease resistance protein
Unigene_345	242	5	167	3	165	46.67	LOC_Os11g15670.1	89	264	8.00E-70	NBS-LRR disease resistance protein
Unigene_346	320	9					LOC_Os12g29710.1	88	111	9.00E-24	NBS-LRR disease resistance protein
Unigene_347	244	5	147	1	147	63.95	LOC_Os07g04900.1	91	317	6.00E-86	NBS-LRR disease resistance protein
Unigene_348	266	8	49	264	216	51.85	LOC_Os02g02640.1	83	121	8.00E-27	NBS-LRR disease resistance protein
Unigene_349	281	6	23	280	258	45.35	LOC_Os06g49360.1	92	309	2.00E-83	NBS-LRR disease resistance protein
Unigene_350	297	7	13	297	285	42.46	LOC_Os12g29690.1	89	361	5.00E-99	NBS-LRR disease resistance protein
Unigene_351	229	5					LOC_Os04g53160.1	87	212	2.00E-54	NBS-LRR disease resistance protein
Unigene_352	1643	81	1518	601	918	42.16	LOC_Os05g50780.1	89	817	0	NBS-LRR resistance-like protein B11
Unigene_353	3696	789	3638	1998	1641	48.14	LOC_Os09g20020.1	91	4038	0	NBS-LRR resistance-like protein B8
Unigene_354	3947	862	3889	2249	1641	48.14	LOC_Os09g20020.1	91	4121	0	NBS-LRR resistance-like protein B8
Unigene_355	3975	856	3917	2277	1641	48.14	LOC_Os09g20020.1	91	4121	0	NBS-LRR resistance-like protein B8
Unigene_356	281	6	134	280	147	48.98	LOC_Os03g37720.1	97	480	1.00E-135	NBS-LRR type disease resistance protein Rps1-k-1
Unigene_357	405	11	149	36	114	39.47	LOC_Os03g37720.1	95	652	0	NBS-LRR type disease resistance protein

											Rps1-k-1
Unigene_358	239	6	109	2	108	69.44	LOC_Os03g37720.1	97	135	5.00E-31	NBS-LRR type disease resistance protein Rps1-k-1
Unigene_359	225	7	105	1	105	76.19	LOC_Os03g37720.1	89	268	5.00E-71	NBS-LRR type disease resistance protein Rps1-k-1
Unigene_360	277	5					LOC_Os12g10180.1	90	351	5.00E-96	NBS-LRR type disease resistance protein Rps1-k-2
Unigene_361	280	7	43	279	237	43.88	LOC_Os12g10180.1	94	424	1.00E-118	NBS-LRR type disease resistance protein Rps1-k-2
Unigene_362	352	10					LOC_Os12g10180.1	94	547	1.00E-155	NBS-LRR type disease resistance protein Rps1-k-2
Unigene_363	402	11	172	402	231	40.69	LOC_Os12g10180.1	95	387	1.00E-106	NBS-LRR type disease resistance protein Rps1-k-2
Unigene_364	372	9	266	3	264	64.77	LOC_Os11g11790.1	91	480	1.00E-134	NBS-LRR type disease resistance protein
Unigene_365	703	21	280	702	423	43.74	LOC_Os11g30210.1	92	581	1.00E-165	NBS-LRR type disease resistance protein
Unigene_366	413	22	350	3	348	41.67	LOC_Os11g30210.1	92	299	2.00E-80	NBS-LRR type disease resistance protein
Unigene_367	834	43	52	834	783	39.21	LOC_Os11g30210.1	90	541	1.00E-153	NBS-LRR type disease resistance protein
Unigene_368	788	49	722	3	720	56.67	LOC_Os11g30210.1	89	888	0	NBS-LRR type disease resistance protein
Unigene_369	266	6	10	264	255	35.29	LOC_Os11g30210.1	94	398	1.00E-110	NBS-LRR type disease resistance protein
Unigene_370	261	5	1	261	261	45.98	LOC_Os11g43320.1	99	502	1.00E-141	NBS-LRR type disease resistance protein
Unigene_371	239	11	29	172	144	51.39	LOC_Os04g39460.1	96	410	1.00E-114	NBS-LRR type disease resistance protein
Unigene_372	228	13	31	228	198	52.53	LOC_Os04g39460.1	95	359	2.00E-98	NBS-LRR type disease resistance protein
Unigene_373	658	24	651	1	651	55.3	LOC_Os04g39460.1	94	1011	0	NBS-LRR type disease resistance protein
Unigene_374	486	13	124	486	363	53.17	LOC_Os04g39460.1	95	696	0	NBS-LRR type disease resistance protein
Unigene_375	508	24	26	232	207	46.38	LOC_Os04g39460.1	97	410	1.00E-113	NBS-LRR type disease resistance protein

Unigene_376	340	7	251	3	249	46.18	LOC_Os07g40810.1	95	547	1.00E-155	NBS-LRR type disease resistance protein
Unigene_377	224	7	208	77	132	46.97	LOC_Os06g06380.1	98	379	1.00E-104	NBS-LRR type disease resistance protein
Unigene_378	479	28	148	438	291	48.45	LOC_Os11g42700.1	92	117	2.00E-25	NBS-LRR type disease resistance protein
Unigene_379	2967	236	2868	1414	1455	46.8	LOC_Os11g38580.1	91	2355	0	NBS-LRR type disease resistance protein
Unigene_380	2874	225	2775	1321	1455	46.8	LOC_Os11g38580.1	90	2030	0	NBS-LRR type disease resistance protein
Unigene_381	2425	224	2168	129	2040	42.79	LOC_Os11g11790.1	94	3768	0	NBS-LRR type disease resistance protein
Unigene_382	322	10	190	2	189	39.15	LOC_Os11g43320.1	100	634	0	NBS-LRR type disease resistance protein
Unigene_383	387	10	194	3	192	43.75	LOC_Os11g43320.1	100	767	0	NBS-LRR type disease resistance protein
Unigene_384	4249	713	63	1649	1587	45.37	LOC_Os11g29990.1	94	4601	0	NBS-LRR type disease resistance protein
Unigene_385	3958	662	63	1649	1587	45.37	LOC_Os11g29990.1	94	4601	0	NBS-LRR type disease resistance protein
Unigene_386	3546	806	3043	311	2733	43.8	LOC_Os11g12050.1	92	3362	0	NBS-LRR type disease resistance protein
Unigene_387	3462	805	3043	311	2733	43.8	LOC_Os11g12050.1	92	3362	0	NBS-LRR type disease resistance protein
Unigene_388	3332	775	3070	311	2760	43.77	LOC_Os11g12050.1	92	3362	0	NBS-LRR type disease resistance protein
Unigene_389	3392	531	17	3073	3057	48.61	LOC_Os11g45930.1	93	2139	0	NBS-LRR type disease resistance protein
Unigene_390	1769	195	17	1768	1752	52.17	LOC_Os11g45930.1	94	1084	0	NBS-LRR type disease resistance protein
Unigene_391	377	8	19	375	357	63.03	LOC_Os04g39460.1	93	410	1.00E-114	NBS-LRR type disease resistance protein
Unigene_392	365	11	194	3	192	43.23	LOC_Os11g30210.1	85	71.9	9.00E-12	NBS-LRR type disease resistance protein
Unigene_393	221	4	200	3	198	41.41	LOC_Os11g38580.1	87	220	9.00E-57	NBS-LRR type disease resistance protein
Unigene_394	213	5	41	211	171	47.95	LOC_Os11g12050.1	87	206	1.00E-52	NBS-LRR type disease resistance protein
Unigene_395	335	11	107	334	228	49.56	LOC_Os06g06380.1	96	571	1.00E-162	NBS-LRR type disease resistance protein
Unigene_396	231	27	228	1	228	42.54	LOC_Os11g12260.1	89	258	4.00E-68	Non-TIR-NBS-LRR type resistance protein
Unigene_397	1317	142	135	344	210	42.38	LOC_Os03g63150.2	89	315	1.00E-84	Powdery mildew resistance protein PM3b
Unigene_398	1226	150	135	344	210	42.38	LOC_Os03g63150.1	90	230	6.00E-59	Powdery mildew resistance protein PM3b
Unigene_399	1141	37	167	481	315	46.98	LOC_Os10g04060.1	92	842	0	Powdery mildew resistance protein PM3b
Unigene_400	1179	41	167	481	315	46.98	LOC_Os10g04060.1	92	842	0	Powdery mildew resistance protein PM3b

Unigene_401	317	7	125	316	192	36.98	LOC_Os10g04060.1	93	468	1.00E-131	Powdery mildew resistance protein PM3b
Unigene_402	280	9	196	2	195	45.13	LOC_Os10g04060.1	91	101	8.00E-21	Powdery mildew resistance protein PM3b
Unigene_403	235	7	51	218	168	42.86	LOC_Os01g06920.1	94	355	3.00E-97	Resistance protein slve1 precursor
Unigene_404	205	4	54	185	132	37.12	LOC_Os01g06920.1	91	250	1.00E-65	Resistance protein slve1 precursor
Unigene_405	924	50	653	3	651	42.4	LOC_Os01g06920.1	86	864	0	Resistance protein slve1 precursor
Unigene_406	600	19	121	600	480	37.71	LOC_Os01g06920.1	88	494	1.00E-138	Resistance protein slve1 precursor
Unigene_407	932	135	153	701	549	57.74	LOC_Os11g11920.1	83	291	1.00E-77	Resistance protein
Unigene_408	741	102	153	731	579	60.62	LOC_Os11g11920.1	82	252	9.00E-66	Resistance protein
Unigene_409	675	41	611	3	609	58.13	LOC_Os08g42700.1	93	924	0	Resistance protein
Unigene_410	1375	166	1315	206	1110	50.72	LOC_Os08g42670.1	90	1332	0	Resistance protein
Unigene_411	314	17	309	43	267	52.06	LOC_Os02g17304.1	90	309	2.00E-83	Resistance protein
Unigene_412	2713	284	2550	1	2550	45.14	LOC_Os02g17304.1	90	3112	0	Resistance protein
Unigene_413	2350	249	2187	1	2187	44.26	LOC_Os02g17304.1	90	2642	0	Resistance protein
Unigene_414	368	13	198	88	111	38.74	LOC_Os02g17304.1	88	105	7.00E-22	Resistance protein
Unigene_415	3586	1679	99	3122	3024	45.83	LOC_Os11g11920.1	96	5031	0	Resistance protein
Unigene_416	3513	1644	644	3049	2406	43.89	LOC_Os11g11920.1	96	4155	0	Resistance protein
Unigene_417	3645	1734	99	3284	3186	46.74	LOC_Os11g11920.1	96	5031	0	Resistance protein
Unigene_418	3572	1699	644	3211	2568	45.13	LOC_Os11g11920.1	96	4155	0	Resistance protein
Unigene_419	438	17	407	3	405	47.41	LOC_Os08g42700.1	94	615	1.00E-175	Resistance protein
Unigene_420	301	9	198	1	198	41.92	LOC_Os02g35210.1	98	553	1.00E-157	Resistance protein
Unigene_421	416	9	36	272	237	45.99	LOC_Os09g34160.1	94	357	1.00E-97	Resistance protein
Unigene_422	323	9	321	10	312	46.47	LOC_Os02g35210.1	95	525	1.00E-148	Resistance protein
Unigene_423	372	14	229	372	144	37.5	LOC_Os01g02810.1	93	272	5.00E-72	Resistance-related receptor-like kinase
Unigene_424	300	8	5	298	294	51.02	LOC_Os01g02840.1	94	61.9	7.00E-09	Resistance-related receptor-like kinase
Unigene_425	357	10	302	24	279	46.59	LOC_Os01g02810.1	81	151	1.00E-35	Resistance-related receptor-like kinase

Unigene_426	220	14					LOC_Os01g02840.1	91	119	3.00E-26	Resistance-related receptor-like kinase
Unigene_427	277	21	73	276	204	47.06	LOC_Os01g02770.1	96	478	1.00E-134	Resistance-related receptor-like kinase
Unigene_428	339	23	61	339	279	42.65	LOC_Os01g02810.1	97	593	1.00E-168	Resistance-related receptor-like kinase
Unigene_429	868	68	597	1	597	43.72	LOC_Os01g02810.1	87	841	0	Resistance-related receptor-like kinase
Unigene_430	1015	528	354	599	246	47.15	LOC_Os01g02360.1	95	65.9	2.00E-09	Resistance-related receptor-like kinase
Unigene_431	1028	521	354	701	348	50	LOC_Os01g02360.1	95	65.9	2.00E-09	Resistance-related receptor-like kinase
Unigene_432	1090	532	354	701	348	50	LOC_Os01g02360.1	95	65.9	2.00E-09	Resistance-related receptor-like kinase
Unigene_433	1077	539	354	599	246	47.15	LOC_Os01g02360.1	95	65.9	2.00E-09	Resistance-related receptor-like kinase
Unigene_434	512	230	268	420	153	57.52	LOC_Os01g02360.1	95	65.9	8.00E-10	Resistance-related receptor-like kinase
Unigene_435	450	219	137	358	222	53.15	LOC_Os01g02360.1	95	65.9	7.00E-10	Resistance-related receptor-like kinase
Unigene_436	510	17	139	510	372	47.85	LOC_Os12g36730.1	81	240	2.00E-62	Stripe rust resistance protein Yr10
Unigene_437	602	38	466	107	360	66.67	LOC_Os10g04342.1	91	165	1.00E-39	Stripe rust resistance protein Yr10
Unigene_438	1557	96	191	1216	1026	50.78	LOC_Os11g37880.1	89	87.7	7.00E-16	Stripe rust resistance protein Yr10
Unigene_439	1654	95	191	1216	1026	50.78	LOC_Os11g37880.1	89	87.7	7.00E-16	Stripe rust resistance protein Yr10
Unigene_440	221	5	14	220	207	38.16	LOC_Os10g04342.1	88	226	2.00E-58	Stripe rust resistance protein Yr10
Unigene_441	425	14	29	424	396	51.52	LOC_Os11g34920.1	97	771	0	Stripe rust resistance protein Yr10
Unigene_442	229	6	213	1	213	56.34	LOC_Os11g34920.1	96	192	2.00E-48	Stripe rust resistance protein Yr10
Unigene_443	249	5	248	3	246	40.24	LOC_Os11g34920.1	97	454	1.00E-127	Stripe rust resistance protein Yr10
Unigene_444	213	6					LOC_Os12g36730.1	95	137	1.00E-31	Stripe rust resistance protein Yr10
Unigene_445	1067	55	891	1	891	41.41	LOC_Os12g17490.1	91	63.9	7.00E-09	Stripe rust resistance protein Yr10
Unigene_446	1779	116	958	1779	822	37.71	LOC_Os11g37880.1	80	593	1.00E-168	Stripe rust resistance protein Yr10
Unigene_447	1154	77	60	974	915	47.54	LOC_Os01g23380.1	95	1810	0	Stripe rust resistance protein Yr10
Unigene_448	798	49	686	3	684	42.69	LOC_Os01g23380.1	95	1273	0	Stripe rust resistance protein Yr10
Unigene_449	202	4	119	15	105	61.9	LOC_Os11g37740.1	88	85.7	3.00E-16	Stripe rust resistance protein Yr10
Unigene_450	516	35	44	514	471	42.46	LOC_Os12g17490.1	98	862	0	Stripe rust resistance protein Yr10

Unigene_451	326	12	10	324	315	47.62	LOC_Os12g17490.1	96	543	1.00E-154	Stripe rust resistance protein Yr10
Unigene_452	1521	113	224	1519	1296	43.9	LOC_Os12g17490.1	91	1094	0	Stripe rust resistance protein Yr10
Unigene_453	550	23	240	548	309	40.78	LOC_Os12g17490.1	95	767	0	Stripe rust resistance protein Yr10
Unigene_454	706	28	240	704	465	37.85	LOC_Os12g17490.1	95	767	0	Stripe rust resistance protein Yr10
Unigene_455	1173	96	224	1171	948	45.46	LOC_Os12g17490.1	91	1273	0	Stripe rust resistance protein Yr10
Unigene_456	1677	118	224	1675	1452	42.63	LOC_Os12g17490.1	91	1094	0	Stripe rust resistance protein Yr10
Unigene_457	394	12	354	1	354	37.29	LOC_Os10g04342.1	86	200	2.00E-50	Stripe rust resistance protein Yr10
Unigene_458	910	36	732	1	732	45.63	LOC_Os11g34920.1	96	1550	0	Stripe rust resistance protein Yr10
Unigene_459	736	28	556	2	555	43.42	LOC_Os11g34920.1	96	1142	0	Stripe rust resistance protein Yr10
Unigene_460	404	10	328	2	327	71.87	LOC_Os01g23380.1	96	680	0	Stripe rust resistance protein Yr10
Unigene_461	205	5	77	205	129	45.74	LOC_Os02g19890.3	99	391	1.00E-108	Stripe rust resistance protein Yr10
Unigene_462	308	6	184	2	183	43.17	LOC_Os02g19890.3	96	511	1.00E-144	Stripe rust resistance protein Yr10
Unigene_463	216	4					LOC_Os06g45690.1	96	54	1.00E-06	Symbiosis-related disease resistance protein
Unigene_464	2435	302	206	2278	2073	42.98	LOC_Os01g08370.1	96	2979	0	Symbiosis-related disease resistance protein
Unigene_465	3671	390	1380	3107	1728	47.63	LOC_Os09g14490.1	94	4234	0	TIR-NBS type disease resistance protein
Unigene_466	3559	369	1268	2995	1728	47.63	LOC_Os09g14490.1	94	4123	0	TIR-NBS type disease resistance protein
Unigene_467	366	5	346	236	111	47.75	LOC_Os09g14490.1	94	131	1.00E-29	TIR-NBS type disease resistance protein
Unigene_468	333	11	216	1	216	54.63	LOC_Os01g06900.1	98	620	1.00E-177	Verticillium wilt disease resistance protein Ve2
Unigene_469	680	21	679	2	678	46.61	LOC_Os01g06900.1	97	1229	0	Verticillium wilt disease resistance protein Ve2
Unigene_470	272	8	260	147	114	43.86	LOC_Os01g06730.1	91	188	4.00E-47	Verticillium wilt disease resistance protein
Unigene_471	541	24	2	541	540	41.3	LOC_Os04g28210.1	88	87.7	2.00E-16	Verticillium wilt disease resistance protein
Unigene_472	240	4	47	199	153	35.95	LOC_Os01g06730.1	94	109	3.00E-23	Verticillium wilt disease resistance protein
Unigene_473	553	16	193	552	360	46.11	LOC_Os01g06730.1	91	636	0	Verticillium wilt disease resistance protein

Unigene_474	223	5					LOC_Os04g28210.1	92	315	2.00E-85	Verticillium wilt disease resistance protein
Unigene_475	401	15	43	399	357	37.54	LOC_Os04g28210.1	94	620	1.00E-177	Verticillium wilt disease resistance protein
Unigene_476	328	7	81	326	246	40.24	LOC_Os01g04070.1	95	539	1.00E-152	Verticillium wilt disease resistance protein
